# Numerical Manifold Method for the Forced Vibration of Thin Plates during Bending

**DOI:** 10.1155/2014/520958

**Published:** 2014-04-30

**Authors:** Ding Jun, Chen Song, Wen Wei-Bin, Luo Shao-Ming, Huang Xia

**Affiliations:** ^1^College of Mechanical Engineering, Chongqing University of Technology, Chongqing 400054, China; ^2^Zhongkai University of Agriculture and Engineering, Guangzhou 510225, China

## Abstract

A novel numerical manifold method was derived from the cubic B-spline basis function. The new interpolation function is characterized by high-order coordination at the boundary of a manifold element. The linear elastic-dynamic equation used to solve the bending vibration of thin plates was derived according to the principle of minimum instantaneous potential energy. The method for the initialization of the dynamic equation and its solution process were provided. Moreover, the analysis showed that the calculated stiffness matrix exhibited favorable performance. Numerical results showed that the generalized degrees of freedom were significantly fewer and that the calculation accuracy was higher for the manifold method than for the conventional finite element method.

## 1. Introduction

According to the concepts of topological manifold and differentiable manifold and based on the discontinuous dynamics of DDA block systems, the numerical manifold method (NMM) adopts the finite cover technique to set up a unified calculation form for the finite element method, the DDA, and the analytical method [1]. NMM combines the advantages of the finite element method and DDA and can deal with various types of complex geometric and material boundaries. Thus, NMM has an obvious advantage in the simulation of large geometric deformations and joint deformations, as well as dynamic and static crossings [2]. The application research on NMM mainly focuses on certain fields, such as geotechnical engineering [3, 4] and crack propagation [5–8]. However, NMM has recently been successfully applied to fatigue failure [9], fluid calculations [10], and seepage [11]. In theoretical research, most NMMs adopt shape functions in the finite element method as the weight functions for the manifold method and use the local cover functions of high-order polynomials to improve the continuity and interpolation accuracy of elements [12–14]. Notably, a polynomial form based on the global coordinate system will increase the calculated condition number of the stiffness matrix or even cause a solution failure. For this reason, Lin et al. [15] improved the form of local cover functions to enhance the computational efficiency and interpolation accuracy of elements. However, the number of generalized degrees of freedom remains large. The mesh-less numerical manifold method is more flexible in the selection of mathematical meshes and can avoid problems resulting from mesh generation [16]. However, plenty of matching points have to be used in the calculation, and matrix inversions have to be intensively performed. Thus, the number of calculations remains large, but the integral accuracy is low, which restricts the application [17, 18]. Recently, Wen and Luo [19] studied the numerical manifold method for polygonal elements, which has a stronger adaptability to physical meshes. The newly developed numerical manifold method based on quadratic B-spline interpolation can further improve the continuity of elements and prevent the linear correlation problem in the conventional numerical manifold method [20, 21].

The discrete finite element solution is the most widely adopted solution for structural dynamics. The interpolation function required by the calculation is based on the static node structure, which is inconsistent with actual problems. The interpolation shape function is related to the element shape and the coordinate system, such that the mesh requirements are relatively high, and the interpolation accuracy is obviously reduced as the structure changes significantly. For the finite elements with mesh structures, the shape functions usually have a low order, which indicates that they cannot meet the requirements of the continuity of displacement by structural deformation. NMM based on finite cover can solve the above problems effectively. The local cover function corresponding to the node displacement of the finite element is no longer relevant to the nodes. If the order of the local cover function is increased, the continuity and interpolation accuracy of the elements can easily be improved. In particular, the manifold method can adopt fixed mathematical meshes that are independent of the physical meshes (material boundary) in setting up the unified interpolation scheme. This method has the advantages of the Lagrangian and Eulerian methods. The manifold method can also avoid problems such as mesh distortion, moving boundary, and migration terms that are difficult to deal with in the finite element and conventional numerical manifold methods [22]. Based on the concept of fixed meshes and considering the deficiency of the existing manifold method in terms of interpolation accuracy, continuity, and solution efficiency, a manifold element with a new cover form and interpolation is constructed. To apply the new element to the solution of structural dynamics, the dynamic theory based on the numerical manifold method is investigated. The numerical manifold scheme for the bending vibration of thin plates is derived, and the final calculation results are comparatively analyzed.

## 2. Basic Principles of NMM

### 2.1. Basic Concept of NMM

**Figure 1:**
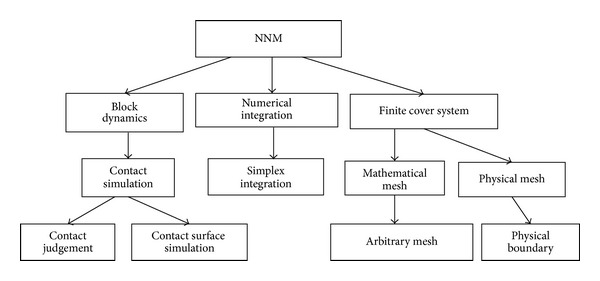
Basic structure of the NMM.

NMM consists of block dynamics, simplex integration, and a finite cover system.  Figure 1 shows the concrete structure [23]. NMM adopts two sets of independent meshes: a mathematical mesh (cover) and a physical mesh (cover). The mathematical mesh is used to define the interpolation accuracy of the solution based on user-defined forms, and the selected mathematical covers can overlap with one another. Consistency with the physical cover is unnecessary, but the mathematical cover has to include the whole material under study. The physical cover is used to describe the geometric properties of the material and the domain of integration, such as the material boundaries, cracks, blocks, and the interfaces of different materials.

In the finite cover system, the intersection of the mathematical cover and the physical mesh is defined as a manifold element; that is, the mathematical cover is subdivided by a physical mesh. The weight function in the manifold method is equivalent to a shape function in the finite element method. If the proper weight function is selected, the weighted summation of the local cover functions can generate the overall cover function of the whole solution domain.

### 2.2. Generation of Overall Cover Function

For simplicity, the manifold method adopts a mathematical cover with a regular structure or a finite element mesh to generate the desired weight function. The generated weight function should meet the property of element decomposition. Meanwhile, the local cover function corresponding to each mathematical cover is not directly relevant to the nodes. This function can be a constant, linear, and high-order polynomial or take other local series forms. The two-dimensional problem can be taken as an example. The local cover function of any mathematical cover *U*_*i*_ is defined as follows:

(1) $$\begin{array}{c} u_{i}\left(x,y\right);\;v_{i}\left(x,y\right)\;\left(x,y\right)\in U_{i}. \end{array}$$

The local cover function (*u*_*i*_, *v*_*i*_) can be expressed as the linear combination of the basis function *f*_*ij*_ with a given *m*-order and the corresponding unknown coefficients *d*_*i*,2*j*−1_ and *d*_*i*,2*j*_. The matrix form is as follows:

(2) {ui(x,y)vi(x,y)}=∑j=1m[fi,j(x,y)00fi,j(x,y)] ×{di,2j−1di,2j}=[fi]Di.

Any manifold element *e* is formed by the intersection of the *q* cover *U*_*e*(*i*)_  (*i* = 1,2,…, *q*) subdivided by a physical cover. The weight function *N*_*e*(*i*)_(*x*, *y*) corresponding to each cover *U*_*e*(*i*)_ is used to solve the overall cover function (*u*, *v*) in the domain, which can be represented as follows:

(3) {u(x,y)v(x,y)} =∑i=1qNe(i)(x,y){ui(x,y)vi(x,y)} =∑i=1q∑j=1m[Ne(i)(x,y)fe(i),j(x,y)00Ne(i)(x,y)fe(i),j(x,y)]  ×{de(i),2j−1de(i),2j}=∑i=1qNe(i)(x,y)[fi](De(i))  =[T]e∙De.

## 3. Two-Dimensional Manifold Method for Cubic B-Spline Interpolation

### 3.1. Definition and Property of the Cubic B-Spline Basis Function

The B-spline basis function can be defined in many ways to facilitate computer programming. In this paper, the reverse derivation method is used to define it [24]. {*x*_1_, *x*_2_,…, *x*_*p*_} is assumed to be a monotonically nondecreasing sequence; that is, *x*_*i*_ ≤ *x*_*i*+1_ and *i* = 0,1,…, *p* − 1, where *x*_*i*_ is the B-spline node. *B*_*i*,*k*_(*x*) is the *i*th *k* B-spline curve with the following definition:

(4) $$\begin{array}{c} B_{i,0}\left(x\right)=\{\begin{array}{cc} 1 & x_{i}\leq x<x_{i+1}\\ 0 & \text{others} \end{array}\\ B_{i,k}\left(x\right)=\frac{x-x_{i}}{x_{i+k}-x_{i}}B_{i,k-1}\left(x\right)+\frac{x_{i+k+1}-x}{x_{i+k+1}-x_{i+1}}B_{i+1,k-1}\left(x\right), \end{array}$$

where [*x*_*i*_, *x*_*i*+1_) is the node interval of *i*th B-spline. During the process of reverse derivation, 0/0 = 0 is required.

B-spline has a local support feature. In other words, when *x* ∉ [*x*_*i*_, *x*_*i*+*k*+1_), *B*_*i*,*k*_(*x*) = 0, when *x* ∈ (*x*_*i*_, *x*_*i*+1_), *B*_*i*,*k*_(*x*) > 0. All B-spline curves in the B-spline node interval [*x*_*i*_, *x*_*i*+1_) meet the property of the element decomposition [19]; that is,

(5) $$\begin{array}{c} \underset{i}{\sum }B_{i,3}\left(x\right)=1. \end{array}$$

According to the definition of the B-spline basis function, the mathematical expression of the cubic B-spline curve expressed in piecewise can be derived as

(6) $$\begin{array}{c} B_{i,3}\left(x\right)=\{\begin{array}{cc} \frac{{\left(x-x_{i}\right)}^{3}}{\left(x_{i+3}-x_{i}\right)\left(x_{i+2}-x_{i}\right)\left(x_{i+1}-x_{i}\right)} & x\in \left[x_{i},x_{i+1}\right)\\ \frac{{\left(x-x_{i}\right)}^{3}}{\left(x_{i+3}-x_{i}\right)\left(x_{i+2}-x_{i}\right)\left(x_{i+1}-x_{i}\right)} & \\ \;+\frac{{\left(x-x_{i+1}\right)}^{3}\left(x_{i+4}-x_{i}\right)}{\left(x_{i+1}-x_{i}\right)\left(x_{i+1}-x_{i+2}\right)\left(x_{i+1}-x_{i+3}\right)\left(x_{i+1}-x_{i+4}\right)} & x\in \left[x_{i+1},x_{i+2}\right)\\ \frac{{\left(x_{i+3}-x\right)}^{3}\left(x_{i+4}-x_{i}\right)}{\left(x_{i+3}-x_{i}\right)\left(x_{i+3}-x_{i+1}\right)\left(x_{i+3}-x_{i+2}\right)\left(x_{i+3}-x_{i+4}\right)} & \\ \;\;+\frac{{\left(x_{i+4}-x\right)}^{3}}{\left(x_{i+4}-x_{i+1}\right)\left(x_{i+4}-x_{i+2}\right)\left(x_{i+4}-x_{i+3}\right)} & x\in \left[x_{i+2},x_{i+3}\right)\\ \frac{{\left(x_{i+4}-x\right)}^{3}}{\left(x_{i+4}-x_{i+1}\right)\left(x_{i+4}-x_{i+2}\right)\left(x_{i+4}-x_{i+3}\right)} & x\in \left[x_{i+3},x_{i+4}\right]\\ 0 & x\notin \left[x_{i},x_{i+4}\right]. \end{array} \end{array}$$

**Figure 2:**
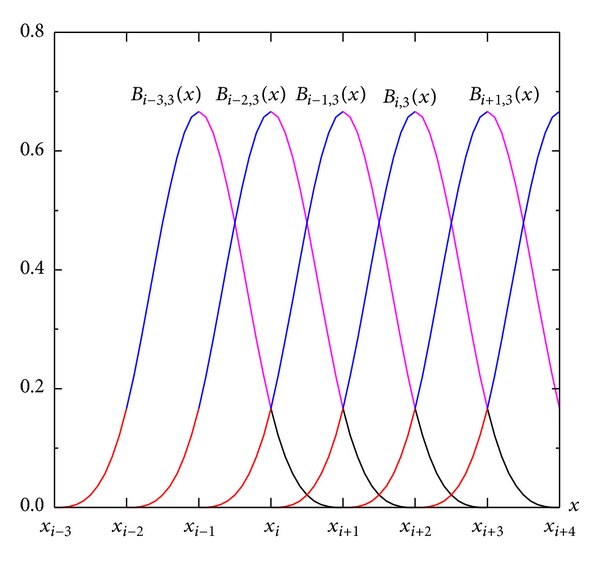
Cubic B-spline curve in piecewise form.

**Figure 3:**
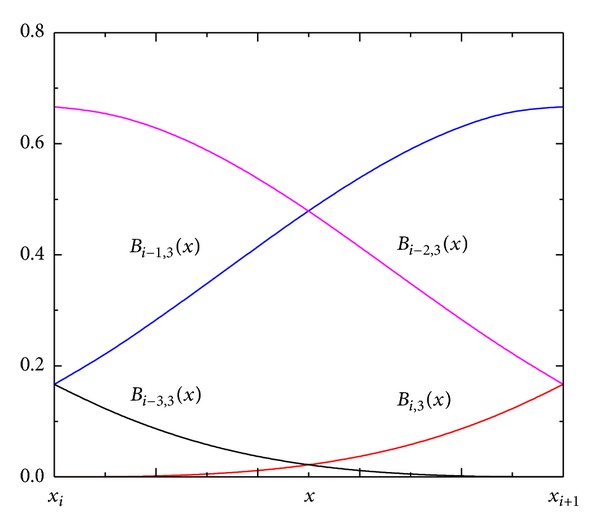
Cubic B-spline curve within the element *i*.

Figure 2 shows the cubic B-spline curves when the B-spline nodes are evenly distributed. Formula (6) can be used to solve all the B-spline curves in the figure.  Figure 3 shows the B-spline curves within the B-spline interval [*x*_*i*_, *x*_*i*+1_) or within the *i* element. The mathematical expression is as follows:

(7) Bi−3,3(x)=(xi+1−x)3(xi+1−xi−2)(xi+1−xi−1)(xi+1−xi),Bi−2,3(x)=(xi+2−x)3(xi+2−xi−1)(xi+2−xi)(xi+2−xi+1) +(xi+1−x)3(xi+2−xi−2)(xi+1−xi−2)(xi+1−xi−1)(xi+1−xi)(xi+1−xi+2),Bi−1,3(x)=(x−xi−1)3(xi+2−xi−1)(xi+1−xi−1)(xi−xi−1) +(x−xi)3(xi+3−xi−1)(xi−xi−1)(xi−xi+1)(xi−xi+2)(xi−xi+3),Bi,3(x)=(x−xi)3(xi+3−xi)(xi+2−xi)(xi+1−xi).

### 3.2. Two-Dimensional Manifold Element of the Cubic B-Spline

A method similar to bilinear interpolation is adopted to construct the B-spline overall interpolation function (as shown in Formula (8)) within the rectangular element domain of *x* ∈ (*x*_*i*_, *x*_*i*+1_) and *y* ∈ (*y*_*j*_, *y*_*j*+1_):

(8) Wi,j(x,y)=[Bi−3,3(x)Bi−2,3(x)Bi−1,3(x)Bi,3(x)] ·[di,jdi,j+1di,j+2di,j+3di+1,jdi+1,j+1di+1,j+2di+1,j+3di+2,jdi+2,j+1di+2,j+2di+2,j+3di+3,jdi+3,j+1di+3,j+2di+3,j+3] ×[Bj−3,3(y)Bj−2,3(y)Bj−1,3(y)Bj,3(y)]=∑i∑jBi−3,3(x)Bj−3,3(y)di,j=Ti,j·Di,j.

In the formula, **D** ^*i*,*j*^ is the generalized displacement vector of the manifold element (*i*, *j*), such that

(9) Di,j=[di,jdi,j+1⋯di+3,j+3]T,Ti,j=[Bi−3,3(x)Bj−3,3(y)Bi−3,3(x)Bj−2,3(y)⋯Bi,3(x)Bj,3(y)].

Each term of **T**_*i*,*j*_ is (**T**_*i*,*j*_)_*k*_, such that

(10) $$\begin{array}{c} \overset{16}{\underset{k=1}{\sum }}{\left(T_{i,j}\right)}_{k}=\overset{3}{\underset{k_{1}=0}{\sum }}B_{i-k_{1},3}\left(x\right)\overset{3}{\underset{k_{2}=0}{\sum }}B_{i-k_{2},3}\left(y\right)=1. \end{array}$$

In the same way, cubic B-spline curves of *B*_*j*−3,3_(*y*), *B*_*j*−2,3_(*y*), *B*_*j*−1,3_(*y*), and *B*_*j*−2,3_(*y*) within *y* ∈ (*y*_*j*_, *y*_*j*+1_) can be obtained in piecewise form. The calculated B-spline functions are substituted into Formula (8) to obtain the final overall cover function of the manifold element.

We suppose that the numbers of manifold elements in the directions of *x*, *y* are, respectively, *N*_1_, *N*_2_, and the corresponding element nodes are *x*_1_, *x*_2_,…, *x*_*N*_1_+1_. Through Formulas (7), a certain number of virtual nodes have to be added to construct the B-spline basis functions. We suppose that the added virtual nodes are *x*_−1_, *x*_0_, *x*_*N*_1_+2_, *x*_*N*_1_+3_; *y*_−1_, *y*_0_, *y*_*N*_2_+2_, *y*_*N*_2_+3_. The selected nodes only need to have the monotonically nondecreasing property.

Based on the continuity of the B-spline surface, the spline surface formed by *k* uniform B-spline basis functions has a *k* − 1-order continuity [25] of *x*, *y* in the element boundary. The above manifold element adopts the model of the generalized node distribution, which is the same as that on the B-spline surface, such that second-order continuity exists in the element boundary.  Figure 3 shows that the B-spline has a desirable property of local support, and its values are small within the interval to ensure the reasonability of the calculated condition number of the stiffness matrix. The number of generalized degrees of freedom required by the calculation is (*N*_1_ + 3)×(*N*_2_ + 3), which is significantly reduced compared with that of the conventional manifold method. Figure 2 shows that the B-spline basis functions have no *δ* property function in the nodes. Thus, these functions can reflect the dynamic property of the structure.

## 4. Manifold Scheme of the Bending Vibration of Simply Supported Thin Plates

### 4.1. Dynamic Equation of the Manifold Element Based on the Principle of Minimum Instantaneous Potential Energy

Suppose that a thin plate has a thickness of *h*, a density of *ρ*, an elasticity modulus of *E*, a bending deflection (displacement) of *W*(*x*, *y*, *t*), an actuating force in the unit area on the plate surface of *q*(*x*, *y*, *t*), a normal direction for the simply supported boundary of *n*, and corresponding fixed deflection and bending moments of ${\overset{-}{W}}_{n}$ and ${\overset{-}{M}}_{n}$. According to the Kirchhoff plate theory [26] and based on the processing method of the boundary condition of the manifold element [1], the principle of the minimum instantaneous potential energy of the numerical manifold of the bending vibration of thin plates can be derived as

(11) ∏G=∬Ω12{κ}T[Df]{κ}dΩ −∬Ω(q−cW˙−ρhW¨)WdΩ +12∫S1β2(Mn−M−n)T(Mn−M−n)dΓ +12∫S1β1(W−W−)T(W−W−)dΓ,

where *Ω* is the area of the thin plate, *S*_1_ is the simply supported boundary, *c* is the damping coefficient, and *β*_1_ and *β*_2_ are the stiffness coefficients of the penalty function. [**D**_*f*_] is the bending stiffness matrix, and {**κ**} is the curvature and torsion of the elastic surface represented by vectors in the directions of *x*, *y* that meets the following equations:

(12) {κ}=[κxκyκxy]T=[−∂2∂x2−∂2∂y2−2∂2∂x∂y]TW={G2}W,

(13) $$\begin{array}{c} \left[D_{f}\right]=\frac{Eh^{3}}{12\left(1-\mu^{2}\right)}\left[\begin{array}{ccc} 1 & \mu & 0\\ \mu & 1 & 0\\ 0 & 0 & \frac{\left(1-\mu \right)}{2} \end{array}\right]. \end{array}$$

*M*_*n*_ in Formula (11) can be expressed as the following equation by the displacement function, where *θ* is the angle from the *x* direction to the *n* direction:

(14) Mn=[(cos⁡⁡θ)2(sin⁡θ)22cos⁡⁡θsin⁡θ] ·[Df]{G2}W={G1}W.

The manifold element of Formula (8) is substituted into Formula (11) along with Formulas (12) and (14) to obtain the discretization form of the minimum instantaneous potential energy:

(15) ∏G∗=∑i=1N1∑j=1N2{∬Ωi,j12({G2}Ti,jDi,j)T[Df]{G2}Ti,jDi,jdΩ+∬Ωi,j(Ti,jDi,j)T×(q−cTi,jD˙i,j−ρhTi,jD¨i,j)dΩ+12∫Si,j1β2({G1}Ti,jDi,j−M−n)T×({G1}Ti,jDi,j−M−n)dΓ+12∫Si,j1β1(Ti,jDi,j−W−)T(Ti,jDi,j−W−)dΓ}.

The first variation is taken from Formula (15) that is the instantaneous variation that satisfies $\delta {\dot{D}}^{i,j}=0$ and $\delta {\ddot{D}}^{i,j}=0$. Therefore, the dynamic equation for any manifold element (*i*, *j*) is given by

(16) $$\begin{array}{c} M_{i,j}{\ddot{D}}^{i,j}+C_{i,j}{\dot{D}}^{i,j}+K_{i,j}D^{i,j}=R_{i,j}, \end{array}$$

where

(17) Mi,j=∬Ωi,jρh(Ti,j)TTi,jdΩ,Ci,j=∬Ωi,jc(Ti,j)TTi,jdΩ,Ki,j=∬Ωi,j({G2}Ti,j)T[Df]{G2}Ti,jdΩ +∫Si,j1(β2({G1}Ti,j)T{G2}Ti,j+β1(Ti,j)TTi,j)dΓ,Ri,j=∬Ωi,j(Ti,j)TqdΩ +∫Si,j1(β1W−(Ti,j)T+β2M−n({G1}Ti,j)T)dΓ.

To facilitate the integration of the stiffness matrix, the transformation matrix **G**_*i*,*j*_ is introduced and can be defined by the following formula, where **D** is the total freedom of degree vector:

(18) $$\begin{array}{c} G_{i,j}D^{i,j}=D. \end{array}$$

The transformation matrix **G**_*i*,*j*_ corresponding to Formula (8) is

(19) $$\begin{array}{c} G_{i,j}={\left[\begin{array}{ccc} 0_{4\times ((i-1)\cdot (N_{1}+3)+j-1)} & I_{4\;\times \;4} & 0\\ 0_{4\times (i\cdot (N_{1}+3)+j-1)} & I_{4\;\times \;4} & 0\\ 0_{4\times ((i+1)\cdot (N_{1}+3)+j-1)} & I_{4\;\times \;4} & 0\\ 0_{4\times ((i+2)\cdot (N_{1}+3)+j-1)} & I_{4\;\times \;4} & 0 \end{array}\right]}_{16\times (\left(N_{1}+3)\cdot (N_{2}+3\right))}, \end{array}$$

where **I**_4  ×  4_ is the identity matrix.

Formula (18) is used to integrate Formula (16) into the overall dynamic equation

(20) $$\begin{array}{c} M\ddot{D}+C\dot{D}+KD=R, \end{array}$$

where

(21) M=∑i=1N1∑j=1N2Gi,jTMi,jGi,j,  C=∑i=1N1∑j=1N2Gi,jTCi,jGi,j,

(22) $$\begin{array}{c} K=\overset{N_{1}}{\underset{i=1}{\sum }}\overset{N_{2}}{\underset{j=1}{\sum }}G_{i,j}^{T}K_{i,j}G_{i,j},\;\;R=\overset{N_{1}}{\underset{i=1}{\sum }}\overset{N_{2}}{\underset{j=1}{\sum }}G_{i,j}^{T}R_{i,j}. \end{array}$$

It can be easily observed that (20) has an analogous form as the overall dynamic equation generated by FEM. Meanwhile, considering that **M** and **K** are symmetric positive matrices, we can solve the natural frequencies of the system by use of the techniques employed in FEM.

### 4.2. Initialization of the Dynamic Equation

Unlike the finite element method, **D**, $\dot{D}$, and $\ddot{D}$ in the manifold method are no longer relevant to the nodes and thus need to be initialized. If the manifold element to be calculated is *N*_1_ × *N*_2_ and the number of degrees of freedom is (*N*_1_ + 3) × (*N*_2_ + 3), the noncoincident points need to be regenerated in the directions of *x*, *y* to obtain the final nodes (*x*_*i*_, *y*_*j*_), *i* = 1,2,…, *N*_1_ + 3, and *j* = 1,2,…, *N*_2_ + 3. If the manifold element corresponding to node (*x*_*i*_, *y*_*j*_) is (*I*_*i*_, *J*_*j*_) and the real initial displacement is initialized with the velocity of *W*(*x*_*i*_, *y*_*j*_, 0) and $\dot{W}(x_{i},y_{j},0)$, we obtain

(23) $$\begin{array}{c} T_{0}D_{0}=W_{0},\\ T_{0}{\dot{D}}_{0}={\dot{W}}_{0}, \end{array}$$

where

(24) T0=[(TI1,J1)|(x,y)=(x1,y2)GI1,J1⋮(TIN1+2,JN2+2)|(x,y)=(xN1+2,yN2+2)GIN1+2,JN2+2(TIN1+3,JN2+3)|(x,y)=(xN1+3,yN2+3)GIN1+3,JN2+3](N1+3)×(N2+3),W0=[W(x1,y1,0)⋯W(xN1+2,yN2+2,0)W(xN1+3,yN2+3,0)]T,W˙0=[W˙(x1,y1,0)⋯W˙(xN1+2,yN2+2,0)W˙(xN1+3,yN2+3,0)]T.

Formulas (23) can be used to obtain the generalized displacement **D**_0_ and the generalized velocity ${\dot{D}}_{0}$, which are substituted into Formula (20) to obtain the generalized initial acceleration:

(25) $$\begin{array}{c} {\ddot{D}}_{0}=M^{-1}\left(R-KD_{0}-C{\dot{D}}_{0}\right). \end{array}$$

### 4.3. Integration Scheme of the Dynamic Equation

When the domain of area integration is a regular rectangular element, Gaussian integration can be adopted. When the domain is an irregular geometric configuration, it can be subdivided into several triangular elements. Then, the simplex integration or Hammer integration is used to obtain the calculation results with sufficient accuracy [27]. When the boundary integration is a regular curve, Gaussian integration can be directly performed. If the boundary integration is a complex curve, it should be subdivided into several, straight-line segments before Gaussian integration is performed.

For time integration, the Wilson-*θ* method can be selected in this paper. Formula (20) is then converted into the linear equation T at time *t* + *θ*Δ*t*. Consider

(26) $$\begin{array}{c} \hat{K}D_{t+\theta \Delta t}={\hat{R}}_{t+\theta \Delta t}, \end{array}$$

where

(27) R⌢t+θΔt=Rt+θΔt+M(6(θΔt)2Dt+6θΔtD˙t+2D¨t) +C(3θΔtDt+2D˙t+θΔt2D¨t),K^=6(θΔt)2M+3θΔtC+K.

After solving **D**_*t*+*θ*Δ*t*_, the generalized total displacement, velocity, and acceleration are solved:

(28) $$\begin{array}{c} {\ddot{D}}_{t+\Delta t}=\frac{6}{\theta^{3}\Delta t^{2}}\left(D_{t+\theta \Delta t}-D_{t}\right)-\frac{6}{\theta^{2}\Delta t}{\dot{D}}_{t}+\left(1-\frac{3}{\theta }\right){\ddot{D}}_{t},\\ {\dot{D}}_{t+\Delta t}={\dot{D}}_{t}+\frac{\Delta t}{2}\left({\ddot{D}}_{t+\Delta t}+{\ddot{D}}_{t}\right),\\ D_{t+\Delta t}=D_{t}+\Delta t{\dot{D}}_{t}+\frac{\Delta t^{2}}{6}\left({\ddot{D}}_{t+\Delta t}+2{\ddot{D}}_{t}\right). \end{array}$$

Formulas (18) and (8) are used to obtain the function of the displacement, velocity, and acceleration of any manifold element (*i*, *j*) at time *t* + Δ*t*:

(29) $$\begin{array}{c} W_{i,j}=T_{i,j}G_{i,j}D_{t+\Delta t}^{i,j},\\ {\dot{W}}_{i,j}=T_{i,j}G_{i,j}{\dot{D}}_{t+\Delta t}^{i,j},\\ {\ddot{W}}_{i,j}=T_{i,j}G_{i,j}{\ddot{D}}_{t+\Delta t}^{i,j}. \end{array}$$

## 5. Calculation Examples and Numerical Analysis

### 5.1. Calculation Example

**Figure 4:**
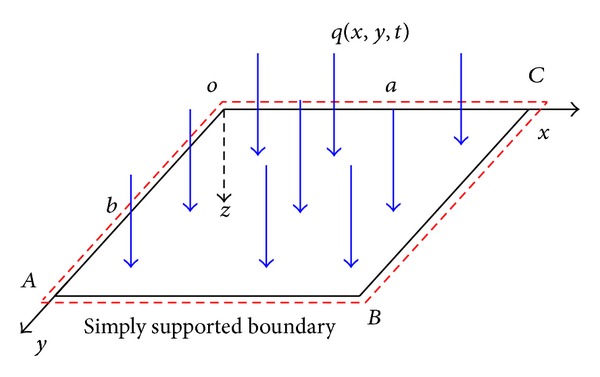
Scheme and configuration of the thin plate.

**Figure 5:**
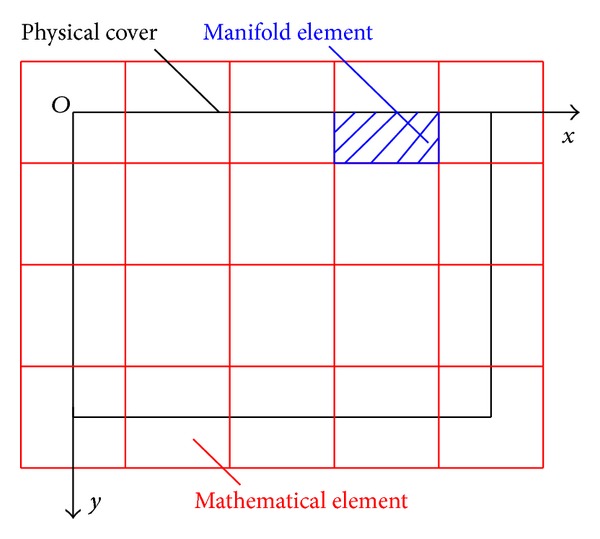
Cover system and manifold element of the thin plate.

As shown in Figure 4, the thin plate that is simply supported on the four edges has a length of *a* = 6.3 m, a width of *b* = 2.1 m, and a thickness of *h* = 1 × 10^−2^ m. The transverse dynamic load is *q*(*x*, *y*, *t*) = *Q*sin⁡(*αt*) Pa, *Q* = 1 × 10^4^ Pa, and *α* = 10. In addition, the elasticity modulus is *E* = 210 GPa, the Poisson's ratio is *μ* = 0.3, the stiffness coefficient of the penalty function is *β*_*i*_ = 1.0 × 10^9^, the material density is *ρ* = 4 × 10^4^ kg/m^3^, the damping coefficient is *c* = 0, and the coefficient of the Wilson-*θ* method is *θ* = 1.4. Different time steps Δ*t* are selected for the calculation. As shown in Figure 5, the fixed rectangular mesh is used as the mathematical cover. The shaded area in the figure denotes any calculated manifold element.

With the theory illustrated in Section 4.1, the generalized instantaneous potential energy for this example can be expressed as

(30) ∏G=∫0b∫0a12{κ}T[Df]{κ}dx dy −∫0b∫0a(q(x,y,t)−cW˙−ρhW¨)Wdx dy +12∫0aβ1(Wy=0)T(Wy=0)dx +12∫0aβ2(Wy=b)T(Wy=b)dx +12∫0bβ3(Wx=0)T(Wx=0)dy +12∫0bβ4(Wx=a)T(Wx=a)dy,

where the latter four terms are the generalized energy form of boundary conditions.

After a large amount of discretization calculation, the matrices **M**, **C**, **K**, and **R** can be calculated by

(31) M=∑i=1N1∑j=1N2(Ci,j)T{∬Ωi,jρh·(Ti,j)TTi,jdx dy}Ci,j,C=∑i=1N1∑j=1N2(Ci,j)T{∬Ωi,jc·(Ti,j)TTi,jdx dy}Ci,j,K=∑i=1N1∑j=1N2(Ci,j)T{∬Ωi,j({G2}Ti,j)T·[Df]·({G2}Ti,j)dx dy}Ci,j +∑i=1N1(Ci,0)Tβ1×{∫Si,01(Ti,0|y=0)T(Ti,0|y=0)dx+∫Si,01β2(Ti,0|y=b)T(Ti,0|y=b)dx}Ci,0, +∑j=1N2(C0,j)T×{∫S0,j1β3(T0,j|x=0)T(T0,j|x=0)dy+∫S0,j1β4(T0,j|x=a)T(T0,j|x=a)dy}C0,j,R=∑i=1N1∑j=1N2(Ci,j)T{∬Ωi,jq(x,y,t)·(Ti,j)Tdx dy},

where {**G**_2_} and [**D**_*f*_] are given in (12) and (13), respectively.

The frequency and the displacement for this case are as follows:

(32) wij=π2(i2a2+j2b2)K0ρh,W(x,y,t)=∑i=1,3,5,… ∑ j=1,3,5,…16QK0π2ij(π4((i2/a2)+(j2/b2))2−(ρh/K0)α2) ·sin⁡iπxasin⁡jπyb(sin⁡(αt)−αwijsin⁡(wijt)).

### 5.2. Numerical Analysis

Table 1 shows the frequency of the manifold method, the accuracy of which will improve along with the increasing density of meshes. The relative error of frequency in the table is defined as *η* = (|*w* − *w*′|/|*w*|) × 100(%). When the 11 × 11 mesh is adopted, the first-order frequency error is only 0.0041%, whereas the fifth-order frequency error is 0.24%.

*W*′, *W*_*t*_′, and *W*_*tt*_′ are defined as the numerical displacement, velocity, and acceleration, respectively. In the same way, *W*_*xx*_′, *W*_*xy*_′, and *W*_*yy*_′ are the partial derivatives of numerical displacement. The corresponding relative errors have the same definitions as in Table 1.  Table 2 shows the velocities and acceleration of the given points at certain time point. The accuracy of the numerical solution is high. Considering that the given interpolation functions has the same continuity and coordination for *x* and *y*, Table 3 only shows part of the partial derivatives of displacement. The numerical solution shows that both stress and strain in the manifold method have good accuracy.

**Figure 6:**
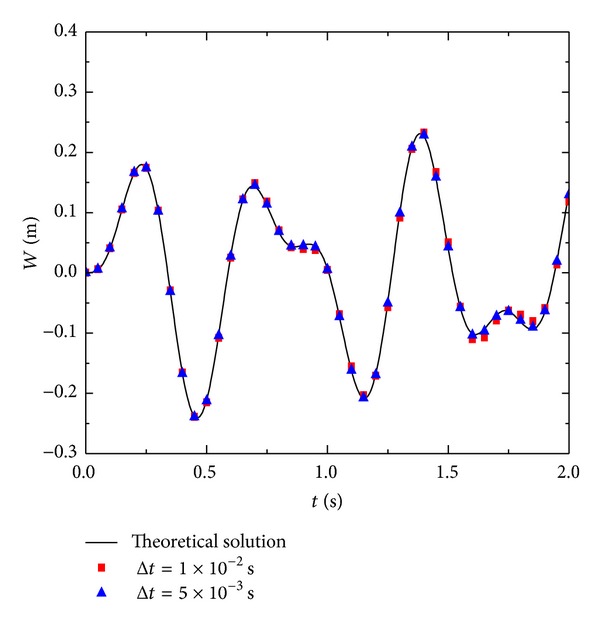
Numerical result of displacement *W*  with 5 × 5 manifold elements.

**Figure 7:**
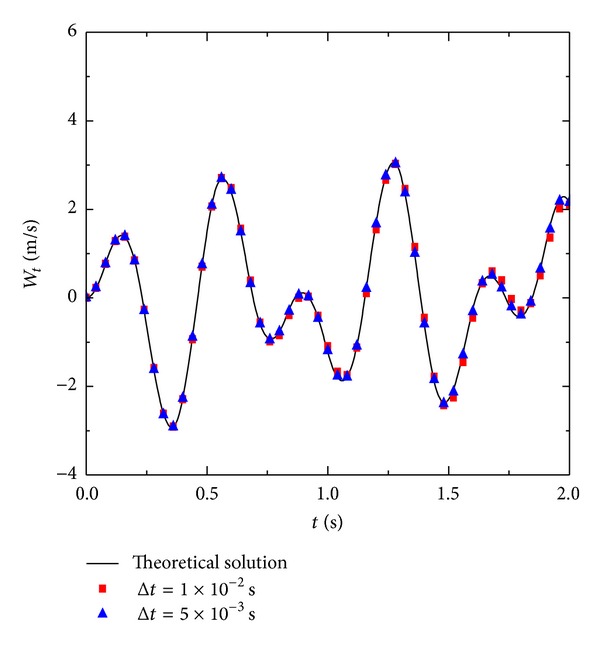
Numerical result of velocity *W*_*t*_ with 5 × 5 manifold elements.

**Figure 8:**
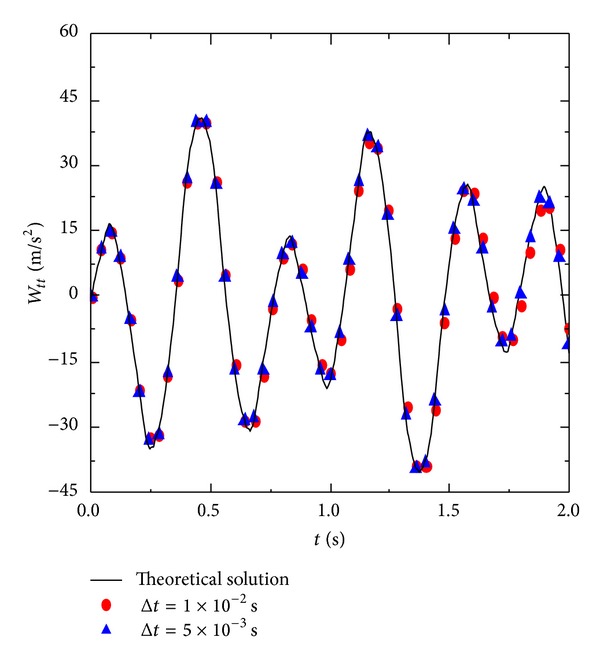
Numerical result of acceleration *W*_*tt*_ with 5 × 5 manifold elements.

**Figure 9:**
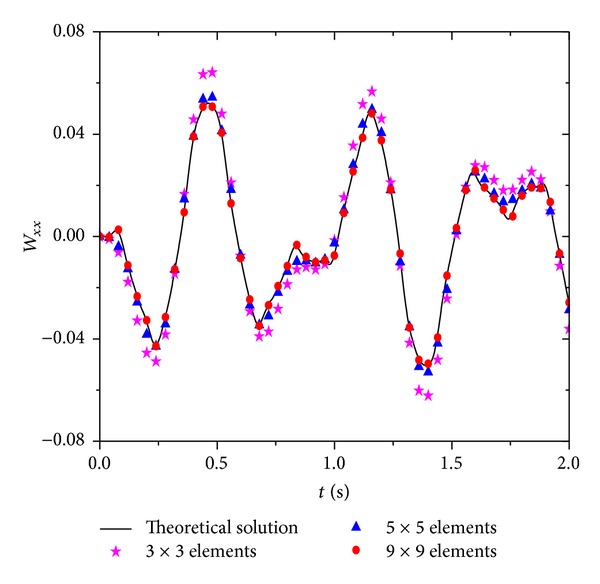
Numerical result of ∂^2^ *W*/∂*x* ^2^ with Δ*t* = 5 × 10^−3^ s.

Figures 6, 7, and 8 are, respectively, the displacements, velocities, and acceleration at the points of *x* = *a*/3 and *y* = *b*/3 for each moment. With the reduction in time step Δ*t*, the accuracy of the numerical solution improves. In particular, with the increase in time steps, smaller step lengths of Δ*t* can significantly improve the accuracy of the numerical solution.  Figure 9 shows some partial derivations at the points of *x* = *a*/3 and *y* = *b*/3. The figure presents the calculated values of the first several periods. As the meshes become denser, the calculation accuracy significantly improves. Therefore, the numerical solution is consistent with the theoretical solution.

Since the manifold method in this paper adopts an overall interpolation approximate, the integrated error norm has to be introduced to reflect the interpolation accuracy fully. $\varepsilon_{0}=(\sqrt{\sum_{k=1}^{N_{a}}{\left(W^{\prime }-W\right)}^{2}}/\sqrt{\sum_{k=1}^{N_{a}}{\left(W\right)}^{2}})\times 100(\%)$ is defined as the integrated error of displacement, where *N*_*a*_ is the number of discrete points equally distributed in the *x* and *y* directions within the thin plate. During calculation, *N*_*a*_ is taken as 11 × 11 discrete points within the thin plate. The integrated errors of the other quantities in Tables 4 and 5 are defined by a similar method.  Table 4 shows the bilinear Hermite finite element. The mathematical software MATLAB is strongly recommended for the calculation, similar to the manifold method. By comparing Tables 4 and 5, the overall degrees of freedom for the manifold method involving the same number of elements are significantly smaller than those for the finite element method, but its calculation accuracy is higher than that of the latter. Furthermore, Table 6 gives the global errors from the cubic B-spline finite element. By comparing Tables 6 and 5, we can safely conclude the cubic B-spline manifold element produces more accurate results than cubic B-spline finite element. All numerical results confirm the validity of the manifold method in solving structural dynamics problems.

## 6. Conclusions

In this paper, based on the nonuniform cubic B-spline basis function, a new numerical manifold method was derived. By using the same structural model as the B-spline surface, the second-order coordinate can be obtained at the boundary of the manifold element. The local support property of the B-spline basis function enables the good performance of the stiffness matrix, which facilitates the numerical calculation. The new method was used to solve the bending problems of thin plates. The linear elasticity dynamic equation was derived based on the principle of the minimum instantaneous potential energy. The method for the initialization of the dynamic equation and the time integration method were also derived. The fixed mathematical mesh was used to implement the manifold method. The numerical results agreed well with the theoretical solution. Compared with the finite element method, the overall degrees of freedom of the cover calculated with the new manifold method were significantly reduced. The calculation accuracy was obviously improved, which indicates the superiority of the manifold method in solving structural dynamics problems. The theoretical and application studies in this paper are preliminary, confined to linear elasticity problems, and limited to situations in which only the time integration method is adopted. Further research on nonlinear problems should be conducted in the future to perfect the theory.

## Figures and Tables

**Table 1:** First five natural frequencies and their relative errors of manifold calculation.

Element	First frequency		Second frequency		Third frequency		Fourth frequency		Fifth frequency	
	*w*_11_′/Hz	*η*	*w*_21_′/Hz	*η*	*w*_31_′/Hz	*η*	*w*_41_′/Hz	*η*	*w*_51_′/Hz	*η*
3 × 3	17.2564	0.084	22.5501	0.60	65.7306	111.79	70.9070	64.50		
5 × 5	17.2427	0.0046	22.4204	0.026	31.2165	0.58	44.9846	4.36	63.9337	9.06
7 × 7	17.2413	0.0035	22.4141	0.0022	31.0619	0.085	43.3865	0.65	60.3066	2.87
9 × 9	17.2411	0.0046	22.4130	0.0071	31.0406	0.016	43.1758	0.16	59.0368	0.71
11 × 11	17.2412	0.0041	22.4126	0.0089	31.0348	0.0026	43.1275	0.052	58.7638	0.24
Theoretical solution	17.2419	0.00	22.4146	0.00	31.0356	0.00	43.1049	0.00	58.6227	0.00

**Table 2:** Velocity and acceleration of the manifold calculation and their relative errors.

Elements	*x* = *a*/3, *y* = *b*/3, Δ*t* = 5 × 10^−3^ s, *t* = 50Δ*t*			
	*W*_*t*_′ (×10^−1^ m/s)	*η*_*t*_	*W*_*tt*_′ (m/s^2^)	*η*_*tt*_
3 × 3	6.2981	0.57	−33.705	1.81
5 × 5	6.2088	1.98	−33.693	1.85
7 × 7	6.2127	1.92	−34.199	0.38
9 × 9	6.2228	1.76	−34.437	0.32
Theoretical solution	6.3340	0.00	−34.328	0.00

**Table 3:** Manifold results of the displacement partial derivatives and their relative errors.

Element	*x* = *a*/3, *y* = *b*/3, Δ*t* = 5 × 10^−3^ s, *t* = 50Δ*t*			
	*W*_*xy*_′ (×10^−2^)	*η*_*xy*_	*W*_*yy*_′ (×10^−1^)	*η*_*yy*_
3 × 3	4.2774	7.85	−4.2777	10.17
5 × 5	3.9085	1.45	−3.8569	0.66
7 × 7	3.9712	0.13	−3.8551	0.71
9 × 9	3.9721	0.15	−3.9208	0.98
Theoretical solution	3.9660	0.00	−3.8827	0.00

**Table 4:** Global errors of the finite method with Hermite element.

Time	*N*_1_ = 7, *N*_2_ = 7, DOFs = 256, Δ*t* = 5 × 10^−3^ s					
	*ε*_0_	*ε*_*t*_	*ε*_*tt*_	*ε*_*xx*_	*ε*_*xy*_	*ε*_*yy*_
20Δ*t*	1.22	0.66	3.21	23.26	9.59	1.52
40Δ*t*	0.34	0.83	2.54	12.50	3.27	0.74
60Δ*t*	0.47	0.47	4.11	6.67	1.41	0.88
80Δ*t*	0.42	0.53	2.49	10.97	2.83	0.78
100Δ*t*	0.50	1.02	2.26	10.25	2.50	0.77

**Table 5:** Global errors of the manifold method.

Time	*N*_1_ = 7, *N*_2_ = 7, DOFs = 100, Δ*t* = 5 × 10^−3^ s					
	*ε*_0_	*ε*_*t*_	*ε*_*tt*_	*ε*_*xx*_	*ε*_*xy*_	*ε*_*yy*_
20Δ*t*	0.55	0.51	3.18	7.51	7.02	0.99
40Δ*t*	0.20	0.83	2.14	4.09	2.47	0.70
60Δ*t*	0.46	0.41	4.12	4.23	1.14	0.84
80Δ*t*	0.36	0.51	1.98	4.28	2.22	0.75
100Δ*t*	0.45	0.94	2.23	2.66	1.87	0.74

**Table 6:** Global errors of the finite method with Cubic B-spline element.

Time	*N*_1_ = 7, *N*_2_ = 7, DOFs = 256, Δ*t* = 5 × 10^−3^ s					
	*ε*_0_	*ε*_*t*_	*ε*_*tt*_	*ε*_*xx*_	*ε*_*xy*_	*ε*_*yy*_
20Δ*t*	1.41	0.77	3.18	12.46	9.59	1.32
40Δ*t*	0.37	0.88	2.14	8.37	3.27	0.72
60Δ*t*	0.58	0.55	4.12	4.69	1.41	0.87
80Δ*t*	0.49	0.56	1.98	6.85	2.83	0.75
100Δ*t*	0.61	1.24	2.23	7.62	2.50	0.76

## References

[1] Pei JM (1997). Numerical manifold method and discontinuous deformation analysis. *Chinese Journal of Rock Mechanics and Engineering*.

[2] Ma G, An X, He L (2010). The numerical manifold method: a review. *International Journal of Computational Methods*.

[3] Zhang G, Zhao Y, Peng X (2010). Simulation of toppling failure of rock slope by numerical manifold method. *International Journal of Computational Methods*.

[4] Ning YJ, An XM, Ma GW (2011). Footwall slope stability analysis with the numerical manifold method. *International Journal of Rock Mechanics and Mining Sciences*.

[5] Zhang HH, Li LX, An XM, Ma GW (2010). Numerical analysis of 2-D crack propagation problems using the numerical manifold method. *Engineering Analysis with Boundary Elements*.

[6] Li SC, Li SC, Cheng YM (2005). Enriched meshless manifold method for two-dimensional crack modeling. *Theoretical and Applied Fracture Mechanics*.

[7] Li SC, Li SC, Zhang JW, Wang ZQ (2007). Numerical manifold method from mathematical theory and its application. *Engineering Mechanics*.

[8] Gao H, Cheng Y (2010). A complex variable meshless manifold method for fracture problems. *International Journal of Computational Methods*.

[9] Wei G, Li K, Jiang H (2010). Incompatible numerical manifold method for fracture problems. *Acta Mechanica Sinica*.

[10] Zhang Z, Zhang X, Yan J (2010). Manifold method coupled velocity and pressure for Navier-Stokes equations and direct numerical solution of unsteady incompressible viscous flow. *Computers and Fluids*.

[11] Jiang QH, Deng SS, Zhou CB, Lu WB (2010). Modeling unconfined seepage flow using three-dimensional numerical manifold method. *Journal of Hydrodynamics*.

[12] Song J, Ohnishi Y (2003). High order rectangular element of manifold method. *Chinese Journal of Rock Mechanics and Engineering*.

[13] Li S, Cheng Y, Wu Y-F (2005). Numerical manifold method based on the method of weighted residuals. *Computational Mechanics*.

[14] Li SC, Cheng YM, Li SC (2006). Meshless manifold method for dynamic fracture mechanics. *Acta Physica Sinica*.

[15] Lin SZ, Qi YF, Su HD (2006). Improved local function of numerical manifold method and its application. *Journal of Yangtze River Scientific Research Institute*.

[16] Gao HF, Cheng YM (2009). Complex variable numerical manifold method for elasticity. *Acta Mechanica Sinica*.

[17] Li SC, Cheng YM (2004). Meshless numerical manifold method based on unity partition. *Acta Mechanica Sinica*.

[18] Gao H, Cheng Y, Jiang H (2010). A meshless manifold method with complex variables for elasticity. *Chinese Journal of Applied Mechanics*.

[19] Wen WB, Luo SM (2012). Polygonal manifold element for thin plate-bending analysis. *Engineering Mechanics*.

[20] Wen WB, Jian KL, Luo SM (2013). 2D numerical manifold method based on quartic uniform B-spline interpolation and its application in thin plate bending. *Applied Mathematics and Mechanics*.

[21] Guo CX, Zeng H (2012). Study on linear dependence problem in high-order numerical manifold method. *Engineering Mechanics*.

[22] Su HD (2011). Study on numerical manifold method with fixed meshes. *Chinese Journal of Theoretical and Applied Mechanics*.

[23] Li SC, Cheng YM (2004). Numerical manifold method and its applications in rock mechanics. *Advances in Mechanics*.

[24] Sevilla R, Fernández-Méndez S, Huerta A (2011). NURBS-Enhanced Finite Element Method (NEFEM). *Archives of Computational Methods in Engineering*.

[25] Piegl L, Tiller W (1996). *The NURBS Book*.

[26] Barber JR (2004). *Elasticity Book*.

[27] Luo SM, Zhang XW, Lu WG, Jiang DR (2005). Theoretical study of three-dimensional numerical manifold method. *Applied Mathematics and Mechanics*.

